# Mechanisms of colorectal liver metastasis development

**DOI:** 10.1007/s00018-022-04630-6

**Published:** 2022-11-27

**Authors:** Tal Shasha, Mandy Gruijs, Marjolein van Egmond

**Affiliations:** 1 Molecular Cell Biology and Immunology, Amsterdam UMC Location Vrije Universiteit Amsterdam, De Boelelaan 1117, Amsterdam, The Netherlands; 2 Cancer Center Amsterdam, Cancer Biology and Immunology, Amsterdam, The Netherlands; 3 Amsterdam Institute for Infection and Immunity, Cancer Immunology, Amsterdam, The Netherlands; 4 Amsterdam UMC Location Vrije Universiteit Amsterdam, Surgery, De Boelelaan 1117, Amsterdam, The Netherlands

**Keywords:** Cancer, Invasion-metastasis cascade, Tumor microenvironment, Epithelial-mesenchymal transition, Circulating tumor cells, Pre-metastatic niche

## Abstract

Colorectal cancer (CRC) is a leading cause of cancer-related death worldwide, largely due to the development of colorectal liver metastases (CRLM). For the establishment of CRLM, CRC cells must remodel their tumor-microenvironment (TME), avoid the immune system, invade the underlying stroma, survive the hostile environment of the circulation, extravasate into the liver, reprogram the hepatic microenvironment into a permissive pre-metastatic niche, and finally, awake from a dormant state to grow out into clinically detectable CRLM. These steps form part of the invasion-metastasis cascade that relies on reciprocal interactions between the tumor and its ever-changing microenvironment. Such interplay provides a strong rational for therapeutically targeting the TME. In fact, several TME constituents, such as VEGF, TGF-*β* coreceptor endoglin, and CXCR4, are already targeted in clinical trials. It is, however, of utmost importance to fully understand the complex interactions in the invasion-metastasis cascade to identify novel potential therapeutic targets and prevent the establishment of CRLM, which may ultimately greatly improve patient outcome.

## Introduction

Colorectal cancer (CRC) is the third most commonly diagnosed cancer and cancer-related cause of death worldwide, accounting for an estimated 1.9 million new cases and 916.000 deaths in 2020 [1]. Of these deaths, it is estimated that 90% is a direct consequence of tumor metastasis [2]. Although the described numbers on this topic vary, it has been reported that up to 50% of patients diagnosed with CRC present with synchronous colorectal liver metastases (CRLM) or develop metachronous CRLM within 5 years after diagnosis [3–6]. Despite technological and surgical advances, long-term survival and cure rates of CRLM patients remain poor [7–9]. Consequently, in the past decade, research regarding CRLM has gained momentum and attempts have been made to understand the steps involved in their establishment, which would allow novel therapeutic strategies. The complexity of the invasion-metastasis cascade comprises a dynamic crosstalk between the tumor and its microenvironment, which facilitates the critical steps of invasion and migration, intravasation, survival in the circulation, extravasation, formation of indolent micrometastases, and finally, successful colonization of the liver parenchyma. This review presents a comprehensive overview of the current knowledge of the mechanisms in the invasion-metastasis cascade, which result in the establishment of CRLM.

### Genetics of CRC

A pioneering model for colorectal tumorigenesis was published in 1990 by Fearon and Vogelstein, in which it was proposed that sequential accumulation of mutations in oncogenes and tumor suppressor genes, such as *APC*, *SMAD4, KRAS*, *BRAF,* and *TP53,* promote progression from normal colonic epithelium to adenoma, and finally, carcinoma [10]. Interestingly, whole exome sequencing (WES) studies revealed that *APC, KRAS,* and *TP53* were among the most frequently mutated genes in CRC and their concomitant CRLM, underlining their importance in CRC oncogenesis and metastasis [11, 12]. Molecular comparisons between the primary tumor and its CRLM have been extensively described elsewhere [13].

A common aberration is the deregulation of the Wnt/β-catenin signaling pathway, which is essential for healthy colonic homeostasis [14]. Mutations in *APC*, a salient regulator of this pathway, are therefore found in the majority of sporadic CRC patients. Hereditary mutations in *APC* give rise to familial adenomatous polyposis (FAP), a rare inherited cancer predisposition syndrome in which patients present with hundreds to thousands of precancerous polyps [7]. Alternatively, patients may exhibit mutations in DNA mismatch repair genes. Defects in the DNA repair apparatus lead to the accumulation of mutations in microsatellite DNA fragments containing repetitive nucleotide sequences, thereby causing microsatellite instability (MSI) [15]. Chronic colonic inflammation, like inflammatory bowel disease, i.e., ulcerative colitis and Crohn’s disease, enhances the risk for CRC development. Chronic inflammation damages the colonic epithelium, causing elevated cell turnover, DNA damage, and consequently an increased chance of mutations, which can eventually lead to colitis-associated CRC (CACRC) [16].

CRC can be classified according to the TNM classification system describing tumor burden (T), presence of tumor cells in sentinel and regional lymph nodes (N), and distant metastases (M). However, patients with the same classification often exhibited heterogeneous drug responses and clinical outcomes. Therefore, an additional classification system has been developed in 2015 by Guinney et al*.*, identifying four consensus molecular subtypes (CMS) in CRC [17]. CMS1 is characterized by MSI, hypermutation, and prominent immune cell infiltration. CMS2 tumors display strong *Wnt* and *Myc* oncogene activation, while the CMS3 subtype exhibits a mixed MSI status, *KRAS* mutations and metabolic dysregulation. Finally, CMS4 has a mesenchymal phenotype with increased transforming growth factor-β (TGF-β) production and pronounced stromal infiltration and angiogenesis [17]. In addition to biological differences, these subtypes differ clinically, with CMS4 representing the most aggressive and metastatic subtype, whereas patients with a CMS1 subtype have the best prognosis [13]. Another prognostic subdivision has been proposed in 2018 by Pagès et al*.* with the use of Immunoscore, describing local densities of CD3^+^ and CD8^+^ cytotoxic T cell infiltration [18]. Patients with high Immunoscores have a lower risk of relapse, whereas a low Immunoscore correlates with a poor prognosis.

## Initiation of malignant transformation

**Fig. 1:**
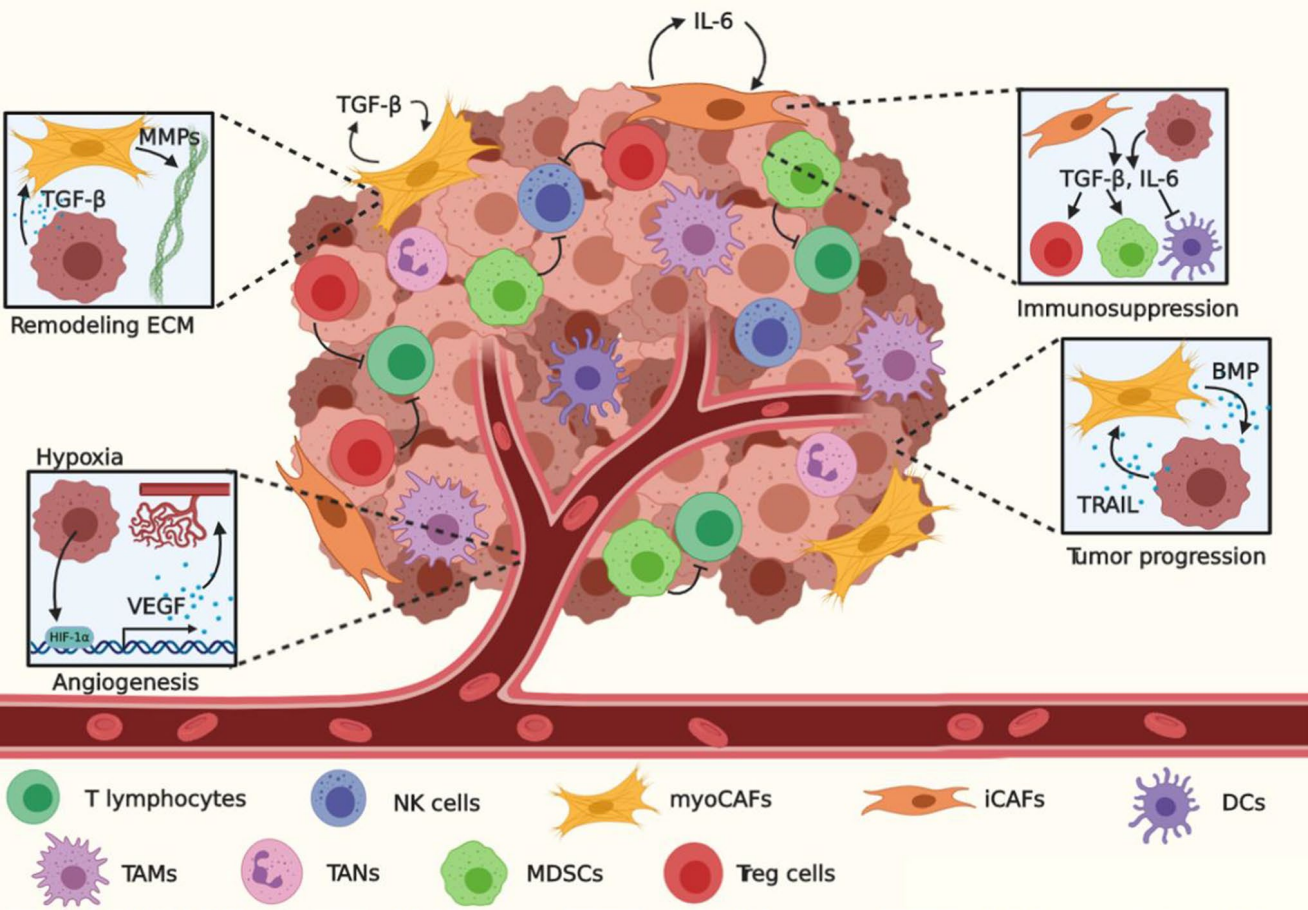
Initiation of malignant transformation. As the tumor grows, hypoxia promotes HIF-1α-dependent VEGF transcription and subsequent angiogenesis. TGF-β secreted by tumor cells and fibroblasts may promote differentiation into myoCAFs, degradation of the ECM through MMPs, and immunosuppression by inducing polarization of T_reg_ cells, M2 macrophages, N2 neutrophils, and MDSCs, and inhibition of NK cells and CTLs. SMAD4^−^ CRC cells may express TRAIL, which triggers CAFs to secrete BMP. In turn, BMP signals through Rho and ROCK to further tumor progression. *NK cells* natural killer cells, *CAFs* cancer-associated fibroblasts, *DCs* dendritic cells, *TAMs* tumor-associated macrophages, *TANs* tumor-associated neutrophils, *MDSCs* myeloid-derived suppressor cells, *T*_*reg*_* cells* regulatory T cells, *MMPs* matrix metalloproteases, *ECM* extracellular matrix, *VEGF* vascular endothelial growth factor, *BMP* bone morphogenic protein

During the process of carcinogenesis, a vast amount of genetically and epigenetically distinct subclones may appear that vary in their capability to survive ever-harshening circumstances, such as increasing spatial and nutritional limitations and immune attack. Selective pressure imposed by the tumor microenvironment (TME) contributes to the natural selection of a few well-adapted clones, which are adequately equipped to progress into advanced or metastatic cancer. Specifically, TME constituents such as hypoxia, immune cells, and fibroblasts are known to be salient regulators of the initial phases of the invasion-metastasis cascade (Fig. 1).

### Hypoxia

Mutations in among others *APC*, *SMAD4, KRAS*, *BRAF,* and *TP53* generate tumor cells that are capable of unlimited proliferation, resulting in an elevated demand for nutrients and oxygen [10]. In addition, excessive growth enhances mechanical stress on the surrounding tissue, which can lead to the collapse of blood vessels, resulting in oxygen deprivation and hypoxia. These hypoxic conditions can lead to an increase in reactive oxygen species (ROS), which damage the tumor cells’ DNA and may cause mutations that further promote malignant transformation. As hypoxia increasingly dominates the tumor, degradation of hypoxia-inducible factor 1-alpha (HIF-1α) is decreased, resulting in the expression of angiogenesis-promoting genes, such as vascular endothelial growth factor (VEGF) and basic fibroblast growth factor (bFGF) [19]. These factors bind their respective receptors on endothelial cells and stimulate their proliferation, migration, and recruitment to the TME to form neoangiogenic blood vessels and restore blood supply to the tumor. These vessels, in turn, are often leaky and instable, leading them to contribute to the vicious circle of chronic tumor-promoting hypoxia.

### Immunosurveillance and immunosuppression

As tumor cells accumulate mutations, they express aberrant antigens on major histocompatibility complex (MHC) class I molecules that can be recognized by the immune system. Immune cells perform extensive immunosurveillance to identify and remove potentially cancerous cells. To prevent elimination, tumor cells in the primary tumor, but also at distant metastatic sites, may suppress and evade immunosurveillance in various manners. First, CRC cells can vastly reduce the expression of MHC class I molecules. Low levels or even the absence of this complex hampers antigen presentation to CD8^+^ cytotoxic T lymphocytes (CTLs), which is indispensable for their activation [20, 21]. MHC class I low-expressing tumors are vulnerable to natural killer (NK) cell-mediated cytolysis through NKG2D. However, many tumor cells upregulate NK cell decoy molecules, which inactivate NK cells after binding. NK cell and CD8^+^ CTL function can further be impeded by the presence of TGF-β, which is well-known for its immunosuppressive actions in the TME [20].

TGF-β is a multifunctional molecule secreted by the tumor, but also by multiple cells in the TME, such as regulatory T (*T*_reg_) cells, myeloid-derived suppressor cells (MDSCs), tumor-associated macrophages (TAMs), and tumor-associated neutrophils (TANs). Besides precluding NK cell and CD8^+^ CTL function, it also polarizes TAMs, TANs, and MDSCs into tumor-promoting cells, with the concomitant secretion of TGF-β to further the establishment of an immunosuppressed TME [22]. Moreover, TAMs that have been polarized into protumorigenic alternatively activated (M2-like) macrophages may also release interleukin (IL)-10, which, together with TGF-β, promotes the development of *T*_reg_ cells [20]. In turn, these cells also contribute to an immunosuppressive TME by producing TGF-β and IL-10, which subsequently inhibits dendritic cell (DC) maturation [20].

MDSCs are a heterogeneous group of myeloid cells, which exhibit many immunosuppressive functions in the developing TME. Under physiological circumstances, immature myeloid cells are generated in the bone marrow under the influence of granulocyte colony-stimulating factor (G-CSF), granulocyte–macrophage colony-stimulating factor (GM-CSF), or macrophage colony-stimulating factor (M-CSF), and differentiate into polymorphonuclear cells (PMN), monocytes, macrophages, or DCs [23, 24]. However, during CRC carcinogenesis, tumors secrete factors, such as TGF-β, prostaglandin E2 (PGE2), IL-6, IL-10, and IL-1β, resulting in the accumulation of MDSCs in peripheral blood and at the tumor site [23].

Although MDSCs likely represent a spectrum of cells with different differentiation stages, two main subgroups have been identified, i.e., monocytic MDSCs (M-MDSCs) and granulocytic MDSCs (G-MDSCs). Despite their phenotypic differences, both subtypes exhibit several common immunosuppressive functions. For example, MDSCs produce ROS and reactive nitrogen species (RNS), which incapacitate CD8^+^ T cells by damaging their T cell receptor (TCR), thereby hampering antigen recognition through MHC class I and subsequent cytotoxic functions [23, 25, 26]. In addition, MDSC-derived RNS can modify CCL2 on CD8^+^ T cells, impairing their chemotaxis to the tumor site [23, 27]. Furthermore, MDSCs have been found to deplete L-arginine, which is an essential amino acid that T cells require for survival and anti-tumor responses [23, 28]. MDSCs can also suppress T cells through TGF-β and IL-10 production and promote polarization of macrophages and PMNs into TAMs and TANs, respectively [23]. Detailed descriptions of MDSC function in CRC have been extensively reviewed elsewhere [23].

Finally, tumor cells themselves can upregulate immunomodulatory membrane proteins to affect immune function. For example, programmed death-ligand 1 (PD-L1) on tumor cells binds to programmed cell death protein 1 (PD-1) on CD8^+^ CTLs, thereby inducing loss of function and exhaustion in the latter [20, 29]. Another checkpoint molecule on tumor cells is CD47, which binds to signal-regulatory protein-α (SIRP-α) on monocytes, macrophages, and neutrophils. This interaction conveys a “don’t eat me” signal to myeloid cells, resulting in their inactivation [30]. Collectively, by employing the processes described above, tumor cells and resident immune cells create an immunosuppressive TME, resulting in immune evasion and malignant transformation with the outgrowth of tumor cells.

### Cancer-associated fibroblasts

As malignant transformation progresses, tumor cells enhance the production of growth factors and cytokines, which maintain tumor growth in an autocrine fashion, but also recruit surrounding stromal cells and induce their differentiation into tumor-promoting cells. The most common stromal cells found in the colorectal TME are cancer-associated fibroblasts (CAFs). Although the exact definition of CAFs remains a matter of debate, typically “cells negative for epithelial, endothelial and leukocyte markers with an elongated morphology and lacking the mutations found within cancer cells” [31], might be considered as CAFs. A number of signaling molecules, including TGF-β, IL-6, and Wnt, have been described to control the plastic transdifferentiation of fibroblasts into two main CAF subtypes termed iCAFs and myoCAFs [31–34]. iCAFs express high levels of IL-6 and exhibit an immunomodulating secretome—hence the prefix. By contrast, myoCAFs, aptly termed due to their resemblance with wound-healing myofibroblasts, exhibit high levels of TGF-β-driven α-SMA expression and have a matrix-producing contractile phenotype. Recently, inhibition of Wnt has been shown to induce a phenotypic switch from myoCAF to iCAF, promoting CRC progression [33]. Interestingly, TGF-β is not only responsible for the myoCAF-associated phenotype; it also inhibits the IL-1 receptor, which normally induces NF-κB signaling and the ensuing IL-6 expression in iCAFs, enabling mutual exclusivity of the two phenotypes [34].

Colorectal CAFs are likely to exhibit the iCAF phenotype, as they have been observed to produce high amounts of IL-6 [33]. This molecule has both pro-inflammatory and anti-inflammatory actions. For instance, IL-6 contributed to the development of CACRC by initiating and perpetuating colonic inflammation [35]. By contrast, it can also inhibit DC maturation and antigen uptake, as well as promote tolerance. Moreover, IL-6 facilitates the recruitment of MDSCs to the TME [20]. These tumor-promoting effects support the rational to therapeutically target IL-6. However, the anti-IL-6 monoclonal antibody Siltuximab did not induce objective clinical responses in patients with advanced and refractory CRC in phase I/II clinical trial [36], suggesting that blockade of a single factor with a dual role in this complex environment is insufficient. Additional roles of IL-6 are also associated with TME modifying effects. For example, IL-6 is a potent inducer of angiogenesis. CRC murine models bearing IL-6-secreting iCAFs with constitutive STAT3 activation exhibited reduced tumor growth after inhibition of angiogenic signaling, suggesting that sustained angiogenesis at least partially depends on IL-6 and STAT3 signaling [37]. A recent study suggested that CAF-secreted IL-6 induced JAK2/STAT3 signaling in CRC cells, leading to the expression of Leucine-Rich Alpha-2-Glycoprotein 1 (LRG1). LRG1, in turn, allowed CRC cells to become more invasive and metastasize to the liver [38].

Another prominent factor secreted by stromal fibroblasts during CRC progression is TGF-β. Its canonical pathway signals through TGFBRI and TGFBRII and promotes the association of SMAD2/3 and SMAD4, which together regulate the expression of TGF-β-related target genes such as VEGF-A and VEGF-C, involved in angiogenesis, and TGF-β itself [39]. Interaction of CRC cells with resident fibroblasts promoted hyperactivated TGF-β1 signaling in the latter, acting in an autocrine manner to create a positive feedback loop stimulating and sustaining the differentiation into myofibroblasts and myoCAFs [39, 40].

It is important to note that tumor-promoting effects of TGF-β mostly occur in late-stage tumors. In the initial phase of CRC tumorigenesis, deletions in tumor suppressor gene *SMAD4* promoted tumorigenesis by precluding functional TGF-β signaling, thus demonstrating the protective nature of the TGF-β pathway in early stage tumors [10, 41]. Paradoxically, loss of *SMAD4* expression has been described to occur typically in later stages of the adenoma to carcinoma sequence, where it is associated with elevated signaling by the TGF-β family, as well as a mesenchymal CRC phenotype, high amounts of stroma, and poor prognosis [10, 42]. This paradox can be explained by an additional member of the TGF-β family, the bone morphogenetic protein (BMP). SMAD4-deficient CRC cells overexpress TRAIL, which stimulates CAFs to secrete BMP [43]. Normally, BMP acts through SMAD4 to exert tumor suppressive effects, but it can also signal in a SMAD4-independent manner. To compensate for the loss of SMAD-4, BMP reverts to Rho signaling via ROCK, resulting in the transcription of genes associated with aggressive transformation [44]. Collectively, the pathways described above represent the first steps of CRC cell differentiation into a more mesenchymal phenotype associated with invasion, motility, and metastasis.

## Epithelial-mesenchymal transition

The established immunosuppressive and tumor-promoting environment is thought to induce epithelial-mesenchymal transition (EMT) or epithelial plasticity [45]. This is a cellular program originally associated with embryogenesis and wound healing that is strongly implicated in invasion and metastasis. During this process, epithelial cells revert from an apical-basal cell polarity toward a front-rear polarity and induce the expression of mesenchymal genes. This metamorphosis is accompanied by dramatic cytoskeletal changes, as well as dissociation of lateral cell–cell junctions, which normally maintain stable epithelial layer integrity. In addition, it promotes mesenchymal morphology and migratory abilities, followed by the invasion of the underlying stroma and subsequent metastasis [45]. Furthermore, cells with epithelial plasticity may become cancer stem cells (CSCs), which exhibit tumor-initiating abilities that are deemed indispensable for the repopulation and seeding of metastatic tumors [46–49]. Cells residing in this state are also endowed with other stem cell-like properties, such as the ability to evade the immune system and resist anti-cancer therapies [46, 50]. As epithelial tumor cells can reversibly adopt mesenchymal cell traits in response to factors in the TME to induce metastasis formation, it is debated whether tumor cells with mesenchymal morphology and CSCs are distinct cell types, or represent the same cell population. Nevertheless, as these cells are thought to promote relapse and metastasis, targeting their stem cell-like traits represents a novel strategy in the treatment of CRC [51]. However, further research is required to identify CSC markers that can unequivocally distinguish these cells from their non-CSC partners [45].

Recent studies have discovered that carcinoma cells exhibiting such epithelial plasticity rarely undergo full EMT. Alternatively, they employ a partial EMT program, yielding cells with both epithelial and mesenchymal traits [45]. It has been well documented that cells executing partial EMT programs exhibit extensive plasticity to modify their phenotype along the EMT spectrum in response to contextual signals [45, 47]. In fact, cells residing in an EMT state can revert back to their initial epithelial phenotype in the scarcely characterized reverse process termed mesenchymal-epithelial transition (MET), which is thought to be required for metastatic colonization in later stages of the invasion-metastasis cascade [47, 52, 53]. As such, hybrid EMT states endow colorectal cancer cells with the highest efficacy for metastasis [45, 54].

Studies investigating genomic alterations between primary tumors and distant metastases revealed consistent homogeneity of functional mutations in driver genes [11, 12], suggesting that EMT is not dependent on DNA mutations, but rather results from epigenetic changes that are imposed on CRC cells by certain environmental contexts. Factors secreted by CAFs, anti-inflammatory immune cells, and tumor cells themselves act in a paracrine or autocrine manner to induce an intracellular signaling cascade resulting in the expression of EMT transcription factors (EMT-TFs), which in turn transcriptionally activate mesenchymal genes. For example, the TGF-β pathway can induce transcription of four main EMT-TF families, i.e., SNAIL, SLUG, ZEB and TWIST. In turn, these transcription factors repress E-cadherin and cytokeratin, and upregulate the mesenchymal adhesion molecule N-cadherin and the structural protein vimentin [45]. Moreover, crosstalk of the TGF-β pathway with the Wnt pathway increases nuclear localization of β-catenin, resulting in the further deconstruction of epithelial cell–cell junctions [55]. EMT-TFs can perpetuate TGF-β signaling by upregulating TGF-β family ligands, constituting a positive feedback loop to sustain the obtained mesenchymal state. Simultaneously, CAFs respond to TGF-β signaling by enhancing the expression of matrix metalloproteinase (MMP)-2 and MMP-9 [40], which are responsible for remodeling the basement membrane and the extracellular matrix (ECM), thereby creating a highway for migrating CRC cells to invade underlying tissues. Interestingly, immune cell-derived factors, such as IL-13, have been shown to promote similar phenotypic shifts in CRC cells [56]. Targeting IL13Rα2, which is often highly expressed on metastatic CRC cells, reversed their invasive phenotype and inhibited the formation of CRLM in vivo [57].

An additional layer of complexity in the regulation of colorectal EMT was revealed by recent advances in single-cell RNA sequencing and functional assays studying microRNAs (miRNAs) and long non-coding RNAs (lncRNAs). One of the most well-established groups of tumor suppressor miRNAs comprises the miR-200 family, which consists of five members dispersed over two genomic clusters (miR-200a/200b/429 and miR-200c/141) [58]. Of these molecules, miR-200c can bind the 3′UTR of ZEB1 to prevent its translation and subsequent EMT [58, 59]. In turn, ZEB1 transcriptionally represses the miR-200 family, constituting a double-negative feedback loop [60]. Clinically, invasive fronts of tumors rarely express miR-200c, resulting in elevated transcription of ZEB1, and concomitant EMT and metastasis [53, 58, 59]. Interestingly, CRLM show an increase in miR-200c compared to the invasive front of the primary tumor, which is concordant with a return to an epithelial phenotype through MET [53].

LncRNAs are thought to interact with miRNAs to induce EMT, but their exact mechanism of action has not yet been elucidated. Nevertheless, it was recently demonstrated that the oncogenic lncRNA H19, which is strongly upregulated in aggressive CRC and associated with poor prognosis, is a potent inducer of EMT in CRC cells [61]. Cell-intrinsic H19 directly interferes with miR-200a and precludes it from suppressing its target genes ZEB1 and ZEB2, thereby promoting their expression and subsequent EMT [61]. In addition, it was shown that non-coding RNAs can affect CRC cells via extrinsic exosomes. In a CACRC model, CAF-derived exosomal lncRNA H19 triggered a CSC phenotype in CRC cells through miR-141 and activation of the Wnt/β-catenin pathway [62]. Mechanistically, H19 acted as a competing endogenous RNA sponge by sequestering miR-141 and precluding it from inhibiting β-catenin and its respective pathway, resulting in CSC formation [62]. These studies suggest that tumor-intrinsic non-coding RNAs, but also stromal cell-derived miRNAs and lncRNAs can promote a mesenchymal stem cell-like phenotype in CRC cells.

After establishing the ability to migrate, CRC cells advance through the underlying stroma where they interact with immune cells before reaching the blood vessels. Novel insights reveal that carcinoma cells with activated EMT programs may upregulate immunosuppressive molecules and repress immunostimulatory membrane complexes to evade potential immunosurveillance [63, 64]. For instance, ZEB1 promotes the expression of PD-L1 via inhibition of its repressor miR-200 [65, 66]. Conversely, EMT has also been reported to function as an NK cell-mediated immune checkpoint. Upon overexpression of SNAIL1, a pivotal EMT-TF, CRC cells increased NKG2D ligand surface expression, rendering them susceptible to NKG2D-dependent NK cell killing [67]*.* Although the exact immunomodulatory mechanisms and contextual signals of EMT programs remain elusive, it is evident that activation of the EMT program fundamentally changes the susceptibility of cancer cells toward immune responses.

While ample evidence supports the role of EMT in tumor progression, an increasing body of studies questions its indispensability for dissemination. One of these paradoxes comprises the notion that tumor cell clusters are more effective at seeding distant metastases than individual cells [68]. The formation of clusters by definition requires the retention of E-cadherin, one of the main epithelial markers repressed during EMT. Conversely, E-cadherin is thought to impede migration, which contradicts the idea that tumor cell clusters can disseminate. Interestingly, these clusters often display a hierarchical organization associated with heterogeneous partial EMT activation: i.e., the cancer cells leading the collective invasion front exhibit a more mesenchymal phenotype, which promotes migration, as well as creating a migratory path by secretion of stroma-remodeling MMPs. Simultaneously, epithelial markers such as E-cadherin are expressed to adhere to the rest of the cluster [47, 69, 70]. Moreover, the leading front cells can interact with CAFs through a heterotypic E-cadherin/N-cadherin adhesion, capitalizing on their mechanical forces to advance through the stroma [71]. By contrast, cancer cells following the leaders display mixed phenotypes of epithelial states and partial EMT, thereby maintaining the integrity of the cluster. In fact, complete loss of E-cadherin impedes metastasis in breast cancer [72], suggesting that retention of the epithelial cell adhesion machinery is required for successful metastasis. Interestingly, invading tumor cell clusters have recently also been reported in CRC, but it remains to be confirmed whether these clusters employ similar mechanisms as described above [73–75].

## Survival in the circulation

**Fig. 2:**
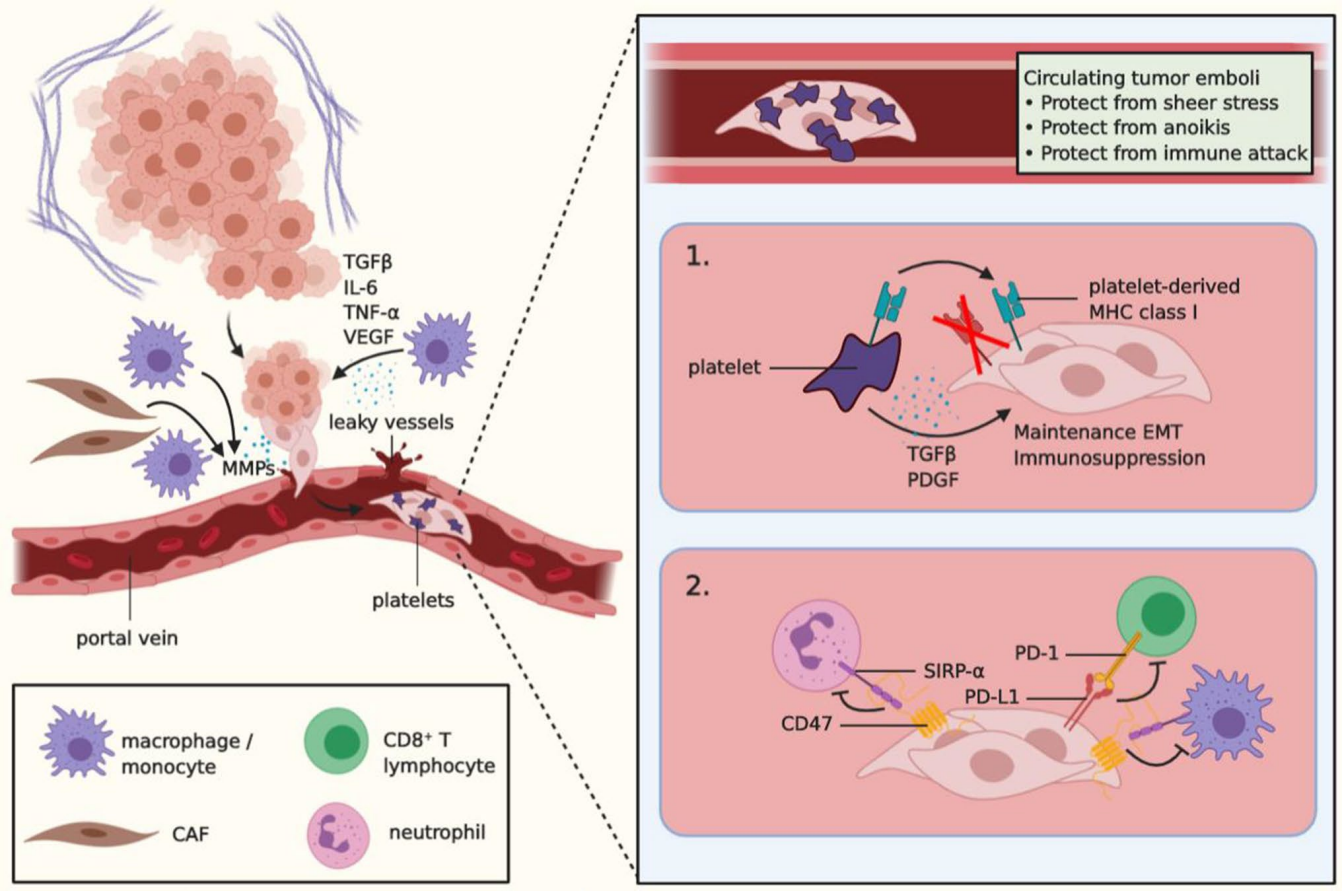
Survival in the circulation. Intravasation of tumor cells into the portal vein is facilitated by TAM-derived signals, such as TGFβ, IL-6, and TNF-α. In addition, TAM-derived VEGF promotes angiogenesis and thus the formation of dysfunctional and leaky vessels, thereby creating a physical entrance for the cancer cells into the circulation. MMPs produced by CAFs, TAMs, and tumor cells can degrade the endothelial basal lamina and cell–cell junctions, which further contributes to intravasation. In the circulation, cancer cells may cluster and form circulating tumor emboli (CTE), which attract platelets and physically shield the cells from sheer stress, anoikis, and immune attack. Furthermore, platelets may produce TGFβ, which promotes maintenance of a more mesenchymal state as well as immunosuppression (1). In addition, platelets may transfer their functional MHC class I complex to tumor cells to further circumvent an immune attack (1). Finally, tumor cells in the circulation may upregulate PD-L1 and CD47, which bind to PD-1 on CTLs, and to SIRP-α on neutrophils and monocytes, respectively, and inhibit these cells from exerting their anti-tumor effects (2). *VEGF* vascular endothelial growth factor, *CAF* cancer-associated fibroblast, *MMPs* matrix metalloproteases, *EMT* epithelial-mesenchymal transition, *MHC* major histocompatibility complex, *PDGF* platelet-derived growth factor, *SIRP-α* signal-regulatory protein-α, *PD-1* programmed cell death protein 1, *PD-L1* programmed death-ligand 1

As CRC cells invade the stroma and navigate toward the tumor vasculature, they may enter the mesenteric or portal circulation (Fig. 2). This process, referred to as intravasation, remains poorly studied. However, it is known that excessive VEGF signaling generates leaky vessels, which may present a physical opening for tumor cells through which they are passively shed into the circulation [76, 77]. Alternatively, MMPs secreted by CAFs, tumor cells, and TAMs may degrade endothelial cell–cell junctions and the basement membrane [77]. Interestingly, TAMs are thought to play an indispensable role in intravasation. In breast cancer, carcinoma cells express epithelial growth factor receptor (EGFR), which, after activation, promotes invasion and migration, and induces secretion of colony-stimulating factor-1 (CSF-1), the main chemoattractant and activator of macrophages. Perivascular macrophages respond to CSF-1 by proliferating and secreting EGF, which in turn further activates breast cancer cells. Thus, this reciprocal interaction yields a positive-feedback loop, facilitating the invasion and intravasation of tumor cells [78]. As CRC cells also express EGFR, similar mechanisms may be involved. In addition, M2-like macrophages that are activated by TGF-β, IL-6, and TNF-α have been found to physically interact with CRC cells, guiding them into the circulation and facilitating distant metastases [79]. A lack of TAMs or their polarization into M1-like macrophages abrogated the formation of distant metastases, suggesting that cancer cells rely on M2 macrophages for dissemination and intravasation [78, 79]. Nonetheless, numerous reports have correlated the presence of M2-like TAMs in CRC with favorable prognosis for patients [80, 81], indicating a more precise characterization of the TME and macrophage phenotype in CRC is required to determine their role in intravasation.

Once in the circulation, circulating tumor cells (CTCs) are exposed to cellular and mechanical threats, impeding their stay in the blood microenvironment. In fact, it is thought that of the millions of cells that are shed into the blood on a daily basis, a mere 0.01% succeeds in forming micrometastases, in part due to the vast elimination of cancer cells after intravasation [47, 82]. One of the first bottlenecks comprises the exposure to shear stress, resulting in the rupture of cells not adequately adapted to these forces [47]. One way to circumvent death by shear stress is to form clusters. Primarily, cluster formation confers a survival advantage to cells in epithelial or partial EMT states by preventing anoikis, a form of cell death triggered in epithelial cells upon detachment from their surroundings [47]. In addition, these circulating tumor emboli (CTE) attract other blood constituents, such as immune cells and platelets, to shield them from their turbulent surroundings, thereby increasing their chances of survival [47].

In addition to protecting CTCs from shear stress, platelets may contribute to CTC survival in several other ways. For example, platelets that coat CTCs form a physical barrier that shields CTCs from immunological attack [83]. Moreover, after binding CTCs, platelets secrete TGF-β and platelet-derived growth factor (PDGF), which maintains the EMT state of tumor cells and contributes to immunosuppression of circulating NK cells [47, 83–85]. In addition, it has been described that platelets can transfer their MHC class I molecules onto CTCs, thereby rescuing them from otherwise imminent NK cell attack due to faulty or missing MHC class I complexes [86]. Furthermore, phenotypic analyses of CRC CTCs have identified CD47 as the predominant molecule upregulated in CTCs compared to the primary tumor, thereby protecting them from attack by myeloid cells [30]. Finally, tumor cells may secrete PD-L1-loaded exosomes into the circulation, resulting in the inactivation and exhaustion of CD8^+^ CTLs [87]. Therefore, further research should aim at targeting determinants of immunosuppression in the blood microenvironment, as well as targeting CTCs to prevent their survival in circulation.

## Extravasation into the liver

**Fig. 3:**
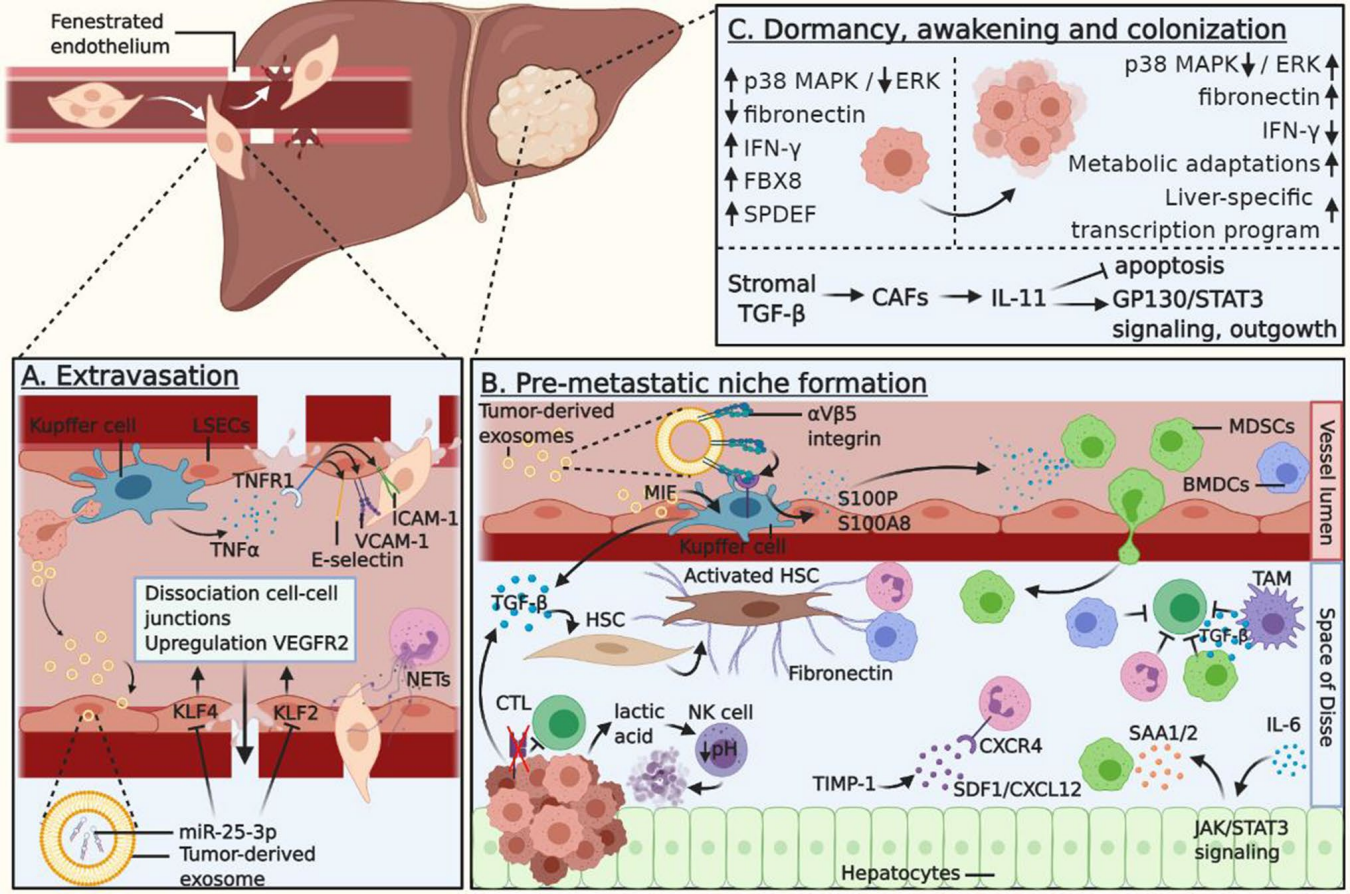
CRLM promoting interactions in the liver. **A** CRC cells navigate through the portal vein and arrive at the fenestrated liver microvasculature, where they extravasate into the liver. Initially, KCs phagocytose the tumor cells. Subsequently, KCs secrete TNFα, which binds to TNFR1 on LSECs and promotes the expression of E-selectin, VCAM, and ICAM-1, facilitating extravasation. Fenestration and leakiness of the vessels can be enhanced by tumor-derived exosomes carrying miR-25-3p, which inhibits KLF4 and KLF2, resulting in dissociation of cell–cell junctions and upregulation of VEGFR2. In addition, neutrophils secrete NETs to capture CTCs in the sinusoids. **B** Tumor-derived exosomes carrying integrin αvβ5 specifically bind to KCs in the liver, resulting in their secretion of S100P and S100A8, which in turn recruit MDSCs to the hepatic pre-metastatic niche. In addition, MIF-bearing tumor-derived exosomes are taken up by KCs, and stimulate TGF-β expression. This TGF-β activates HSCs and promotes the production of fibronectin, which attracts neutrophils and BMDCs. Presence of TIMP-1 results in increased SDF1/CXCL12 levels, which promote CXCR4-dependent neutrophil recruitment. Furthermore, IL-6-induces JAK/STAT3 signaling in hepatocytes. These release SAA1 and SAA2, which in turn recruit MDSCs. Recruitment of these cells suppresses the hepatic pre-metastatic niche. For example, TGF-β secreted by MDSCs and TAMs, as well as neutrophils and BMDCs, inhibit CD8^+^ CTL function. In addition, tumor cells downregulate MHC class I and upregulate NK cell decoy molecules to evade immune surveillance. Lactic acid released by tumor cells can decrease intracellular NK cell pH, resulting in apoptosis of and thus evasion from these cells. **C** Dormant cells exhibit high p38 MAPK and low ERK levels, as well as reduced fibronectin in the ECM and elevated signaling through IFN-γ, FBX8 and SPDEF. CRC cells that awaken and grow out into overt metastases colonizing the liver exhibit low p38 MAPK and high ERK levels, as well as high fibronectin in the ECM and decreased IFN-γ signaling. CRC cells activate liver-specific transcription programs and metabolically adapt to the liver. TGF-β-dependent IL-11 production by CAFs inhibits apoptosis, and supports outgrowth through activation of GP130/STAT3 signaling. *LSECs* liver sinusoidal endothelial cells, *TNFα* tumor necrosis factor α, *TNFR1* tumor necrosis factor receptor 1, *ICAM-1* intracellular adhesion molecule 1, *VCAM-1* vascular cell adhesion molecule 1, *KLF* Krüppel-like Factor, *MIF* macrophage migration inhibitory factor, *MDSC* myeloid-derived suppressor cell, *BMDC* bone marrow-derived cell, *TIMP-1* TIMP metallopeptidase inhibitor 1, *SAA* serum amyloid A, *HSC* hepatic stellate cell, *IFN-γ* interferon-γ, *FBX8* F-box only protein 8, *SPDEF* SAM Pointed Domain Containing ETS Transcription Factor

The fraction of CRC CTCs that survives the harsh conditions of the blood microenvironment will become entrapped in the liver microvasculature. In these capillaries, various cells interact to aid CRC CTCs in traversing the endothelial wall and entering the liver parenchyma, a process termed extravasation (Fig. 3a). Although it remains unclear which exact cellular and environmental mechanisms govern this step of the invasion-metastasis cascade, several reciprocal interactions between cancer cells, their exosomes, and the hepatic microenvironment have been proposed to play a key role in facilitating entrance to the liver.

Extravasation begins when CTCs are arrested in the liver microvasculature. These capillaries, referred to as sinusoids, are lined with liver sinusoidal endothelial cells (LSECs) and macrophages (i.e., Kupffer cells; KCs), which are organized in a fenestrated structure facilitating passive extravasation of entrapped CTCs [88]. Interestingly, vascular permeability may be enhanced by tumor-derived exosomes carrying miR-25-3p [89]. When taken up by LSECs, CRC-derived miR-25-3p inhibits the transcription factors KLF4 and KLF2, resulting in downregulation of the endothelial cell–cell junction proteins ZO-1, occludin, and Claudin-5, whereas VEGFR2 is upregulated, respectively [89]. Moreover, KCs can phagocytose exosomes containing angiopoietin-like protein 1 (ANGPLT1), which normally decreases MMP9 expression. However, CRC-derived exosomes contain remarkably low levels of ANGPLT1, resulting in upregulated MMP9 expression and subsequent degradation of the vasculature [90]. Collectively, these changes in expression promote fenestration of the endothelium, thereby further facilitating extravasation.

Interactions between CTCs, LSECs, and KCs have also been implicated in promoting extravasation. Initially, after the arrest of CRC cells in the sinusoids, KCs can eliminate CTCs by phagocytosis [91]. However, the rapid influx of CTCs into the liver parenchyma induces a pro-inflammatory cascade in LSECs and KCs, promoting the polarization of the latter toward a protumorigenic phenotype [92–94]. Initial extravasation stimulates KCs to release TNF-α, which binds its respective receptor TNFR1 on LSECs and promotes endothelial expression of the vascular adhesion receptors E-selectin, vascular cell adhesion molecule-1 (VCAM-1), and intercellular adhesion molecule-1 (ICAM-1) [93]. The binding of these receptors to their cognate ligands on CRC CTCs induces diapedesis and transendothelial migration, promoting successful extravasation into the liver parenchyma [94]. Interestingly, E-selectin ligands sialyl Lewis x, sialyl Lewis a, and CD44v6, a colorectal CSC marker, have been correlated in the clinic with poor prognosis and metastatic progression [88, 95, 96], suggesting that targeting these glycoproteins might decrease extravasation and therefore mitigate CRLM formation.

Finally, neutrophils have also been described to enhance CRC CTC arrest in liver sinusoids. For example, by adhering to CTCs, they function as a scaffold to facilitate extravasation [97]. In addition, activated neutrophils secrete neutrophil extracellular traps (NETs), which consist of released DNA molecules that normally entangle pathogens in response to an infection, but are now deployed to capture tumor cells in the sinusoids to promote metastasis [98]. Neutrophils have also been described to secrete MMPs after tumor cell arrest, further facilitating extravasation [47, 99]. In conclusion, various resident and circulating cells aid CRC CTCs in entering the liver, where the pre-metastatic hepatic niche is being prepared.

## Preparation of pre-metastatic niche

Following successful extravasation, one of the main challenges is the reciprocal adaptation of tumor cells and the novel hepatic microenvironment. As the primary tumor develops, it coevolves with its TME into a tumor-promoting and immunosuppressive entity, which allows CRC progression. However, after arrival in the liver, CRC cells encounter a plethora of activated immune cells as well as various parenchymal and non-parenchymal liver cells that lack such supportive signals as found in the primary TME, impairing the survival of cancer cells in this novel and hostile organ. Nevertheless, in a series of steps that are not yet well understood, tumor cells and tumor-derived exosomes may recruit immunosuppressive cells and remodel the hepatic microenvironment, forming a so-called pre-metastatic niche that contains fertile soil for colonization of the liver by arriving CTCs (Fig. 3b).

The formation of the hepatic pre-metastatic niche is an early event, occurring prior to the arrival of CRC CTCs. The existence of such a supportive niche implies that CRC exhibits organotropism, i.e., that there is a preference to metastasize to specific organs, rather than representing a stochastic event. Although CRC organotropism to the liver can be explained by the evident anatomic position of the portal vein, carrying blood directly from the gut to the liver, recent studies have highlighted an elegant mechanism, in which CRC-derived extracellular vesicles carrying nucleic acids and integrins fuse specifically with KCs in the liver, altering their gene expression and promoting the formation of a pre-metastatic niche. For example, it has been described that CRC-derived exosomes expressing KC-specific integrin αvβ5 were phagocytosed by KCs, resulting in the expression of the pro-inflammatory proteins S100P and S100A8 [100]. These molecules, in turn, initiated pre-metastatic niche formation in the liver by recruiting MDSCs [101]. In addition, CRC-derived extracellular vesicles carrying miR-21-5p promoted the synthesis of IL-6 in KCs and a concomitant pro-inflammatory microenvironment in the liver [102]. Similarly, CRC-derived exosomes containing miR-135a-5p were released from a hypoxic primary TME, which led to enhanced adhesion of CTCs in the liver through upregulation of the LATS2-YAP-MMP7 axis in KCs, as well as local suppression of CD4^+^ T cells through the CD30-TRAF2-p65 axis [103]. A recent study suggested that CRC-derived exosomes containing HSPC111 reprogrammed lipid metabolism in hepatic stellate cells (HSC), resulting in increased CXCL5 secretion that recruited CTCs through the CXCL5-CXCR2 axis [104].

Moreover, the interaction of liver-tropic integrins with the hepatic microenvironment may alter the ECM, advancing the formation of a pre-metastatic niche. For example, integrin α5β1 is expressed on CRC cells and can engage with fibronectin, the most abundant hepatic ECM glycoprotein [105, 106]. Following this binding, α5β1 induces intracellular focal adhesion kinase (FAK) signaling, which results in the expression of integrin α2β1 and its respective binding to ECM protein collagen I, securing its position within the liver microenvironment [105]. Recent studies suggested that citrullination of collagen I enhances the adhesion of CRC CTCs to the ECM, which might promote colonization through MET [107]. Collectively, these interactions are likely to play a key role in determining liver-specific metastases in CRC and may therefore represent novel therapeutic targets to prevent the establishment of CRLM.

CRC cells must also overcome potential functional immunosurveillance in the liver. It was shown that CRC cells may evade NK cells, the most abundant type of immune cell in the liver, through the production of lactic acid [108]. Elevated lactic acid concentrations in NK cells decreased their intracellular pH, resulting in ROS-dependent mitochondrial dysfunction and apoptosis [108]. Furthermore, it was demonstrated that epithelial NOTCH1 signaling in the tumor induced TGF-β2 expression in the hepatic pre-metastatic niche, resulting in TGFBR1-dependent neutrophil recruitment to the liver [109]. In turn, neutrophils inhibited CD8^+^ CTLs, thereby contributing to immunosuppression in the pre-metastatic niche [109].

In addition to the inhibition of resident cytotoxic cells, further suppression of the hepatic pre-metastatic niche can be achieved through the recruitment of bone marrow-derived cells. For example, tumor-derived exosomes containing increased levels of macrophage migration inhibitory factor (MIF) were shown to educate KCs and promoted TGF-β production [110]. Consequently, HSCs became activated and secreted fibronectin, which stimulated the migration of bone marrow-derived macrophages and granulocytes to the liver [110]. Enzymes involved in ECM remodeling, such as tissue inhibitor of metalloproteinases (TIMP)-1 may also contribute to the development of the hepatic pre-metastatic niche by enhancing hepatic SDF-1/CXCL12 levels, resulting in CXCR4-dependent neutrophil recruitment [111]. Furthermore, parenchymal hepatocytes released serum amyloid A1 (SAA1) and SAA2 after IL-6-mediated JAK/STAT3 signaling, which subsequently recruited MDSCs to the liver pre-metastatic niche [112]. Other chemokines such as CXCL1, CXCL2, and CCL2 can be secreted by TAMs, KCs, LSECs, and activated HSCs in the premetastatic liver, resulting in the recruitment of MDSCs that promoted CRC cell survival by suppressing CD8^+^ CTLs and inducing T_reg_ cells [88]. Other mechanisms independent of innate or adaptive immune responses have also been observed [88, 113, 114]. Collectively, reciprocal interactions between different liver cell populations are essential in the establishment of an immunosuppressive hepatic pre-metastatic niche.

## Cellular dormancy

The final challenge comprises long-term survival and growth in the distant organ. After the successful invasion of the liver pre-metastatic niche, CRC cells may immediately grow out into overt metastases. However, in some cases, CRC cells may enter a quiescent state in which they further adapt and survive in the changing environment (Fig. 3c). Dormant cells alter their metabolism and halt proliferation, giving rise to a phenomenon referred to as cellular dormancy, which results in overall cessation of tumor growth. Alternatively, tumor mass dormancy may occur as a result of immune-mediated surveillance or ineffective delivery of nutrients and oxygen, maintaining these micrometastases small and thus clinically undetectable [115]. Dormant cells, or cells that have detached from the primary tumor at later stages, may eventually grow out and cause metastases weeks, months, or even years later. Although dormancy neither is a required step in metastasis development, nor does it always occur, it is of utmost clinical importance to understand the mechanisms governing the entrance into cellular dormancy, the maintenance of this state, and the subsequent reactivation and metastatic outgrowth of dormant cells to improve detection and treatment of minimal residual disease.

The microenvironmental and cell-intrinsic cues triggering cellular dormancy in CRC have scarcely been described, as most work has been performed in breast and pancreatic cancer and their concomitant metastases to the bone. Nevertheless, several cellular processes, cytokines, and signaling pathways, especially the mTOR pathway, have been identified as key regulatory contributors of dormancy in disseminated tumor cells [116]. Most importantly, a p38 MAPK^high^/ERK^low^ ratio is the main indicator of cellular dormancy [115–117]. Diminished extracellular signaling due to low fibronectin levels or downregulation of growth factor receptors prevents ERK signaling and upregulates p38 MAPK, which in turn further inhibits ERK and promotes G0-G1 arrest and subsequent dormancy [115–117]. Furthermore, activation of p38 MAPK in response to nutritional stress induces ATF6α-dependent upregulation of mTOR signaling, resulting in dormancy and subsequent survival of tumor cells [118]. TGF-β family members TGF-β2 and BMP7 may activate p38 MAPK, which results in cyclin-dependent kinase (CDK)-4 inhibition and transcription of p21 and p27, leading to cell cycle cessation and cellular quiescence [119, 120]. Recently, FBX8 has been found to promote MET and CRC cell dormancy in the liver by directly binding CDK4, c-Myc, and HIF-1α, leading to ubiquitin-dependent degradation of these proteins and inhibition of cell cycle, proliferation, angiogenesis, and metastasis [121]. In addition, SAM pointed domain containing ETS TF (SPDEF) was shown to impede transcription of Wnt-related cell cycle genes by the disrupting binding of β-catenin to its transcription factors TCF1 and TCF3, causing cellular dormancy in CRC [122]. Dormancy may also be induced in tumor cells by microenvironmental cues such as low fibronectin and collagen I density, as well as interferon-γ secreted by CD4^+^ cells or CD8^+^ CTLs [116]. Finally, autophagy has been implicated in both promoting and terminating cellular dormancy, rendering its exact role in this process inconclusive [123]. In short, several pathways may induce cellular dormancy in tumor cells, but as these mechanisms might be tumor-type specific, it is important to verify them and further investigate dormancy in models for CRLM.

## The awakening and outgrowth

In the last and most lethal phase of the invasion-metastasis cascade, CRC cells residing in the liver may awaken from dormancy and grow out into clinically detectable CRLM (Fig. 3c). Nonetheless, the vast majority of cells will succumb to elimination in this novel environment or will not awake from their dormant state, rendering colonization the most rate-limiting and inefficient process in the invasion-metastasis cascade [82, 124]. Most experimental models used to study colonization are osteotropic and pneumotropic models, which lack a dormant phase. Consequently, the escape from dormancy and ensuing metastatic colonization has not yet been adequately characterized. Nevertheless, it is becoming increasingly clear that for successful colonization in fibroblast-rich organs, rigorous remodeling of the ECM is required. These mechanisms often rely on integrin signaling, the release of MMPs and angiogenesis, and have been extensively described elsewhere [125]. In addition, recent studies have shed light on metabolic adaptations that disseminated CRC cells must undergo to grow out into macroscopic metastases.

It was recently reported that CRC cells experienced a dramatic epigenetic switch from a colon-specific gene transcription program to a liver-specific gene transcription program [126]. For instance, hepatocyte growth factor (HGF) may activate a liver-specific cholesterol metabolic pathway, allowing adaptation to the liver and subsequent outgrowth of CRLM [127]. Metabolic differences between CRC cells and the liver may also be overcome by the utilization of creatinine and adenosine triphosphate (ATP) from the hepatic environment to generate phosphocreatine, a high-energy compound often reserved for rapid ATP synthesis, which fuels tumor growth and metastasis to the liver [128]. Moreover, the liver environment can metabolically reprogram CRLM through upregulation of aldolase B, which shifts the cells’ metabolism toward fructose metabolism, thereby promoting outgrowth [129]. Alternatively, signals from the hepatic microenvironment may promote escape from dormancy. For example, CCL7 secreted by monocytic MDSCs may bind to CCR2 on dormant CRC cells, thereby activating JAK/STAT3 signaling, resulting in outgrowth and colonization [130]. Similarly, GP130/STAT signaling may be induced by IL-11 derived from TGF-β-stimulated CAFs, which suppresses apoptosis and promotes outgrowth into CRLM [131]. These liver-specific examples represent some of the few described adaptations required for successful colonization of the liver and clearly illustrate the need to study these organ-specific interactions in further detail.

## Therapeutic approaches

Surgery remains the hallmark treatment for primary CRC. However, it was shown in animal models that abdominal surgery may promote CRLM development through adherence to CTCs in the liver [132, 133]. As such, it was proposed to treat patients preoperatively with anti-EGFR monoclonal antibodies (mAb), which may eliminate CTCs and reduce the risk of CRLM development [134, 135]. Alternatively, blocking adhesion molecules such as E-selectin was shown to decrease tumor cell adhesion in the liver and subsequent CRLM [136–138]. In addition, neoadjuvant chemotherapy, radiofrequency ablation (RFA), or microwave ablation can be used to treat CRLM [7, 13]. When patients have CRLM with wild-type *KRAS* status, patients may be eligible for treatment with anti-EGFR mAbs, as this will block EGF binding, and induce growth arrest. However, when patients have CRLM with mutated EGFR signaling pathways, anti-EGFR mAbs are ineffective [139]. Alternatively, FOLFOX regimens in combination with bevacizumab, a VEGF inhibitor, have also entered the clinic, making it one of the first treatments targeting the TME [140]. The introduction of Immunoscore and the recent CMS classification has emphasized the role of distinct cellular, molecular and genetic markers in CRC, leading to significant changes in therapeutic approach and prognosis [17]. MSI tumors contain a high mutational burden and have been found to respond exceptionally well to immune checkpoint blockade, rendering it the CRC subtype with the best prognosis [141–144].

## Future directions

Progression in the invasion-metastasis cascade from CRC to CRLM results from dynamic interactions between the tumor and its ever-changing TME. The mechanisms described above illustrate that immune cells, CAFs, and other TME-specific constituents play an indispensable role in CRLM development, thereby providing a strong rationale for targeting the TME in combination with cytotoxic anti-tumor therapies [145]. A major advantage of this approach comprises the genetic stability of cells in the TME, which lack extensive heterogeneity as found in tumor cells, thereby minimizing the chance of therapeutic resistance. Moreover, resistance mechanisms originating in the TME, such as placental growth factor (PGF) production after anti-VEGF therapies [146, 147], will also be impeded through such a treatment [145].

Although this concept is attractive, the main question remains which TME constituents represent suitable targets. Directing treatment at the TME implies targeting healthy cells, which may result in strong toxicity due to off-target effects. Alternatively, inhibition of TME constituents that are key regulators of EMT, angiogenesis, and immunosuppression, may attenuate tumor progression [39]. One example is immune checkpoint blockade, which can relieve immunosuppression in the TME and induce durable responses in multiple tumor types. The use of immune checkpoint inhibitor (ICI) pembrolizumab, as well as nivolumab in combination with ipilimumab, has been approved for the treatment of a subgroup of patients with metastatic MSI CRC, while many other ICIs are currently under investigation in clinical trials [142, 143, 148, 149]. Unfortunately, many CRC patients are unresponsive to ICI treatment, most probably due to resistance mechanisms related to persisting immunosuppression [142, 150, 151]. Alternative immune checkpoint molecules, tumor-intrinsic interferon-γ signaling, and TGF-β-dependent exclusion of CD8^+^ CTLs have been proposed as resistance mechanisms in multiple tumors, but colorectal-specific resistance mechanisms remain to be elucidated [152–154].

Several studies in mice have successfully resensitized immune cells to ICI. For example, a recent study demonstrated that enforced expression of relaxin, an anti-fibrotic peptide, was able to reverse the fibrotic liver environment and reinvigorate resident immune cells [155]. Treatment of CRLM with relaxin and anti-PD-L1 therapy produced a highly synergistic anti-metastatic effect, significantly inhibiting CRLM progression and prolonging survival in mice [155]. Further research should assess the safety and efficacy of such combination therapies in CRLM patients. In addition, it was demonstrated that murine CRC models with elevated levels of TGF-β-activated stroma display T cell exclusion, which cannot be reversed with anti-PD-1/PD-L1 immunotherapy [152]. However, T cell infiltration into the tumor was accomplished after TGF-β inhibition. This promoted a Th1 phenotype and diminished tumor burden, suggesting the use of TGF-β inhibitors together with immune checkpoint blockades in the clinic for CRC patients expressing high levels of TGF-β [152]. M7824, an anti-PD-L1/TGF-β-RII fusion protein, is currently under investigation in a phase Ib/II clinical trial for patients with metastatic CRC (NCT03436563). It is important to note, however, that inhibition of TGF-β may also promote the formation of novel malignancies, as its pro-apoptotic effects in healthy tissues are abrogated [39]. Therefore, the development of therapeutic strategies targeting tumor stroma should be guided by robust biomarkers that can monitor malignant transformation and progression.

Recently, studies targeting stromal factors in mice have identified promising therapeutic targets that may revolutionize the treatment of CRLM. For instance, the TGF-β coreceptor endoglin is highly expressed on activated endothelial cells and CAFs, and its expression was associated with poor prognosis in CRLM [156]. TGF-β signaling through this receptor promoted angiogenesis, as well as CAF invasion and tumor metastasis. Specific targeting of endoglin by neutralizing antibody TRC105 was shown to stabilize blood vessels and reduce CAF invasion, thereby decreasing the metastatic burden of CRLM [156]. In addition, combined therapy of TRC105 with anti-PD-1 antibodies in pre-clinical CRC models was shown to significantly enhance therapeutic efficacy [157]. TRC105 is currently investigated as a single agent and in combination with bevacizumab in phase II and III clinical trials for patients with advanced cancers [158, 159] (NCT01332721; NCT00582985).

Another promising target is CXCR4, the receptor for SDF-1/CXCL12, which is expressed in CAFs, MDSCs and cancer cells [160]. Binding of SDF-1/CXCL12 to CXCR4 on CAFs promotes fibronectin production as well as immunosuppression and exclusion [160, 161]. Hence, elevated CXCR4 levels in human CRLM are associated with poor prognosis and resistance to immunotherapy [160, 162]. Accordingly, blockade of CXCR4 with small molecule CXCR4 antagonist AMD3100 (Plerixafor or Mozobil) in metastatic breast cancer mitigated desmoplasia, enhanced CD8^+^ CTL infiltration, and relieved immunotherapy resistance [161]. A phase I clinical trial is currently assessing the impact of AMD3100 on the TME in patients with advanced CRC (NCT02179970).

In conclusion, the liver is the most common site of metastasis in patients with CRC, and yet, there are many questions that remain unanswered regarding the establishment of CRLM. Nevertheless, it is evident that the TME is vital for malignant progression in the invasion-metastasis cascade, as it coevolves with the tumor, and contributes to the selection of plastic clones that are able to readily adapt to their novel environments, causing the lethal effects of metastatic disease. Therefore, unraveling the biology of cellular plasticity is key to understanding and predicting tumor progression, development of distant metastases, and resistance to therapy. We live in a new era of cancer research, in which big data and OMICS approaches, such as epigenomics, metabolomics, and transcriptomics have the potential to unveil the unfathomable complexity of the invasion-metastasis cascade, and with that, advance the development of novel therapies to prevent, and hopefully cure, CRLM.

## Data Availability

Not applicable.
